# Earlier than expected introductions of the *Bemisia tabaci* B mitotype in Brazil reveal an unprecedented, rapid invasion history

**DOI:** 10.1002/ece3.8557

**Published:** 2022-01-23

**Authors:** Jorge R. Paredes‐Montero, Muriel Rizental, Eliane Dias Quintela, Aluana Gonçalves de Abreu, Judith K. Brown

**Affiliations:** ^1^ 8041 School of Plant Sciences The University of Arizona Tucson Arizona USA; ^2^ Facultad de Ciencias de la Vida Escuela Superior Politécnica del Litoral ESPOL Guayaquil Ecuador; ^3^ Federal University of Goiás Goiânia Brazil; ^4^ EMBRAPA Rice and Beans Santo Antônio de Goiás Brazil

**Keywords:** dispersal, invasive species, migration, museum collections, whitefly

## Abstract

During 1991, in Brazil, the presence of the exotic *Bemisia tabaci* B mitotype was reported in São Paulo state. However, the duration from the time of initial introduction to population upsurges is not known. To investigate whether the 1991 B mitotype outbreaks in Brazil originated in São Paulo or from migrating populations from neighboring introduction sites, country‐wide field samples of *B*. *tabaci* archived from 1989–2005 collections were subjected to analysis of mitochondrial cytochrome oxidase I (mtCOI) and nuclear RNA‐binding protein 15 (RP‐15) sequences. The results of mtCOI sequence analysis identified all *B*. *tabaci* as the NAFME 8 haplotype of the B mitotype. Phylogenetic analyses of RP‐15 sequences revealed that the B mitotype was likely a hybrid between a B type parent related to a haplotype Ethiopian endemism (NAFME 1–3), and an unidentified parent from the North Africa‐Middle East (NAF‐ME) region. Results provide the first evidence that this widely invasive B mitotype has evolved from a previously undocumented hybridization event. Samples from Rio de Janeiro (1989) and Ceará state (1990), respectively, are the earliest known B mitotype records in Brazil. A simulated migration for the 1989 introduction predicted a dispersal rate of 200–500 km/year, indicating that the population was unlikely to have reached Ceará by 1990. Results implicated two independent introductions of the B mitotype in Brazil in 1989 and 1990, that together were predicted to have contributed to the complete invasion of Brazil in only 30 generations.

## Graphical abstract

Two independent introductions of the B mitotype in Brazil, in 1989 and 1990, were predicted to have contributed to the complete invasion of Brazil in only 30 generations.

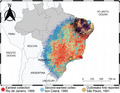

## 1 INTRODUCTION

The whitefly *Bemisia tabaci* (Gennadius) (Hemiptera: Aleyrodidae) consists of an unknown number of mitochondrial groups (Brown, 2010; De Moya et al., 2019), herein “mitotypes,” based on the genetic variability of the cytochrome oxidase subunit I (COI) sequence. Several mitotypes have been previously referred to as biotypes, races (see references in: Brown, 2010), or proposed to represent distinct “species” (Bellows et al., 1994; Dinsdale et al., 2010) belonging to one of at least seven major phylogeographical clades (Brown, 2010). Recently, phylogenomic analysis of 2184 single‐copy orthologous genes (de Moya et al., 2019) was shown to recapitulate the latter phylogeographic‐mitochondrial clades, and corroborated the existence of at least five lineages (Brown, Coats, et al., 1995; Brown, 2010; Gill & Brown, 2009; Hadjistylli et al., 2016; de Moya et al., 2019), which corresponded to a taxonomic rank above biotype/race, mitotype, and previously proposed cryptic “species.” Most mitotypes of *B*. *tabaci* comprise haplotypes that are relatively benign within their native habitat(s), however, several B‐, Q‐like and other haplotypes have become invasive pests and plant virus vectors in agroecosystems in tropical‐subtropical and some warm‐temperate regions of the world (Bayhan et al., 2006; Brown, 2010; Chu et al., 2006; Dennehy et al., 2006; Masood et al., 2017; Papayiannis et al., 2008; Paredes‐Montero et al., 2019, 2020; Ueda & Brown, 2006; Viscarret et al., 2003).

In Brazil, a 1994 report suggested that the introduction of the B mitotype (biotype or referred to by some as MEAM species) occurred in 1991 (Lourenção & Nagai, 1994). Despite the significance of the invasion, the identity of the suspect B mitotype has not been verified beyond a provisional identification, which was based on silverleaf symptoms observed in whitefly‐infested squash plants (*Cucurbita* spp.) (Lourenção & Nagai, 1994). The development of silverleaf symptoms in squash plants within the Americas has been a reliable indicator of B‐mitotype invasions (Brown et al., 1992; Schuster et al., 1991; Yokomi et al., 1990). The silverleaf phenotype was first associated with whitefly‐infested squash in Israel during the 1980s (Burger et al., 1983), suggesting that B mitotype was endemic there and possibly to other nearby locales. The proposed endemism of the B mitotype to northeast coastal Africa and the Middle East (NAF‐ME) has been corroborated by analyses of protein polymorphisms, molecular markers (RAPDs, microsatellites), and DNA sequence data (mtCOI; nuclear genes) (Brown, 2010; Hadjistylli, 2010; Hadjistylli et al., 2016).

The invasion and colonization of Brazil (2,100,000 mi^2^; excluding the Amazon rainforest) by the B mitotype of *B*. *tabaci* has not been well‐studied, with estimates suggesting that it occurred in about 6 years post‐introduction in 1991 (Oliveira et al., 2000). However, there is great potential for the extensive colonization to have occurred earlier than 6 years owing to the arid subtropical climate there, particularly, during the dry seasons, and the widespread cultivation of agricultural crops that are reproductive hosts. Together, these conditions are favorable to short generation times and rapid demographic expansion under barrier‐free conditions (Bethke et al., 1991; Butler et al., 1983; Mansaray & Sundufu, 2009; Quintela et al., 2016).

In Brazil, the rapidly increasing population sizes and dispersal of *B*. *tabaci* throughout the invaded region in 1991, reminiscent of a “lag phase” (Mooney & Cleland, 2001; Sandlund et al., 2001), are consistent with the early invasion stages experienced elsewhere during invasions of the exotic B and Q mitotypes (Banks et al., 2001; Brown, Coats, et al., 1995; Butler et al., 1989; Chu et al., 2006; Costa & Brown, 1991; Gunning et al., 1995; Luo et al., 2002; Martinez‐Carrillo & Brown, 2007; Perring et al., 1992; Price et al., 1987). Although the first recognition of the B mitotype in Brazil was in Sao Paulo in 1991, the duration from the arrival time to population upsurges is not known. Characterizing whitefly populations collected before 1991 is crucial to determine sites of introduction, the timeframe from arrival times to explosion into invasiveness, and pathways of invasion in Brazil.

Computer simulations of the demography of invasive insect species can aid in identifying local ecological factors that were critical to the establishment of an invasive insect so that effective mitigation measures can be identified and implemented (Coutts et al., 2018; Excoffier et al., 2009; Lawson Handley et al., 2011; Sandlund et al., 2001). Models that consider the geographical and environmental complexity of landscapes, the geographic positions (sites) of the invasion(s), and life history parameters of the target insect species have been used for modeling short‐ and long‐distance dispersal in temporal and spatial terms. For example, the SPLATCHE software (Currat et al., 2019) can facilitate modeling of the migration of the B mitotype, and is particularly useful for accounting for dispersal constraints in the context of unique environmental and geographic characteristics (Currat et al., 2019).

In this study, adult whitefly from an archived collection of *B*. *tabaci*, available in Brazil were analyzed using a mitochondrial and nuclear molecular marker. Whitefly collections from Brazil represented field samples collected immediately before and after the proposed widespread invasion of the B mitotype in 1991 (Lourenção & Nagai, 1994). Mitotype identification of *B*. *tabaci* was carried out by polymerase chain reaction (PCR) amplification and analysis of the 3′‐end of the mitochondrial cytochrome oxidase I (mtCOI‐3′) with a global reference mtCOI database available in the J.K. Brown laboratory, University of Arizona. The nuclear RNA‐binding protein 15 (RP‐15) gene was PCR‐amplified and sequenced, and analyzed for nucleotide divergence and phylogenetic relationships. The spatio‐temporal patterns of dispersal for the B mitotype was simulated using SPLATCHE 3 (Currat et al., 2019), assuming a normal population growth.

## 2 MATERIALS AND METHODS

### 2.1 Whitefly DNA extraction and sequencing

Adult *B*. *tabaci* whiteflies were collected live from cultivated and uncultivated plant species in 35 different locations in Brazil during 1989–2005. Whitefly adults were transferred to a glass vial containing 70–95% ethanol and stored at −20°C. The whiteflies were cataloged in the EMBRAPA Genetic Resources and Biotechnology (CENARGEN) insect collection maintained in Brasília, Brazil (Table S1). Two *B*. *tabaci* samples were obtained from the JK Brown laboratory collection (University of Arizona), EN8 from Israel, and Eth3 from Ethiopia, previously shown to group phylogenetically with B mitotype haplotypes NAFME 8 and 3, respectively (Paredes‐Montero et al., 2021) (Table S1). Three other whitefly samples from the JK Brown laboratory collection were included for pairwise comparisons or to root the tree, a sample belonging to the A mitotype (New World species) from Ecuador, and two from Uganda (courtesy, Dr. James Legg, IITA Tanzania), one belonging to the Q mitotype, and the other identified as the ‘Uganda‐Sweet potato’ outlier.

Total DNA was extracted and purified using an optimized protocol (Paredes‐Montero et al., 2019). Briefly, individual female whiteflies were transferred to a sterile microfuge tube containing 25 µl of nuclease‐free water followed by overnight incubation at room temperature. Female (2*n*) whiteflies were selected for all but one sample (40399), which contained only males (1*n*). Using a flat wood toothpick (Diamond^®^, Fishers, IN), individual whiteflies were transferred to a sterile 1.5 ml microfuge tube containing 20 µl CTAB lysis buffer (100 mmol/L Tris‐HCl pH 8.0, 20 mmol/L EDTA pH 8.0, 1.4 mol/L NaCl, 0.2% 2‐mercaptoethanol, and 2% hexadecyltrimethylammonium bromide) and ground with a micropestle. The pestle was washed with 600 µl of CTAB lysis buffer to keep all whitefly fragments into the buffer. Proteinase K was added at 0.005 mg/ml, and samples were incubated overnight at 55°C, followed by incubation at 65°C for 15 min to inactivate the Proteinase K. One volume of chloroform was added, and the mix was centrifugated (Eppendorf Model 5415R, Eppendorf, Hamburg, Germany) at 16,000 g for 3 min at 4°C for phase separation. One volume of 100% isopropanol and 40 µg glycogen were added to the supernatant, with incubation for 10 min at 4°C. Total nucleic acids were pelleted by centrifugation at 16,000 *g* at 4°C for 10 min. The pellet was washed with 70% ethanol, air‐dried, and dissolved in 10 mmol/L Tris‐HCl, pH 8.0.

The mtCOI‐3′ fragment of ~1015 base pairs (bp) consisting of the 3′‐end of the mtCOI and contiguous tRNAleu of 66 bp was amplified by PCR from samples collected in Brazil and from two of the eight B mitotype haplotypes of *B*. *tabaci*, EN8 (NAFME 8) and Eth3 (NAFME 3). The previously published NAFME 1–2 and 4–7 reference sequences (Paredes‐Montero et al., 2021) were downloaded from the GenBank database (Table S1). The mtCOI fragment was amplified with primers F‐COI‐628‐5′‐GATCGAAATTTTAATAGATCTTTTTATGATCC‐3′ and R‐COI‐1629‐5′‐TGTTCTATTGTAAAACTAGCACTATTTTG‐3′ (Paredes‐Montero et al., 2020). Each reaction contained 12.5 μl of 1× Jumpstart REDTaq ReadyMix (Sigma‐Aldrich), primers (0.4 μM each), 20 ng whitefly DNA, and double‐distilled water to a final volume of 25 μl. Cycling parameters were as follows: initial denaturation at 94°C for 5 min, followed by 35 cycles at 94°C for 20 s, 53°C for 20 s, and 72°C for 1 min, with a final extension at 72°C for 20 min. The amplicons were visualized by agarose gel (1%) electrophoresis in 1× TAE buffer, pH 8.0, containing 1× gel red (Biotium) at 100 V for 50 min.

The RP‐15 fragment of 773 bp was amplified by PCR from *B*. *tabaci* samples collected in Brazil and from two of the eight haplotype references for *B*. *tabaci*, EN8 (NAFME 8), and Eth3 (NAFME 3). The RP‐15 sequences for NAFME1‐2 and 4–7 were not available for comparison. The RP‐15 fragment was amplified by PCR using the primer pair F‐RP15‐190–5′‐CACCCACTTCATCCTCACCC‐3′ and R‐RP15‐962–5′‐TCACCCCAGGCATCATAAGC‐3′. Cycling parameters were initial denaturation of 95°C for 1 min, followed by 30 cycles of 95°C for 15 s, 55°C for 15 s, 72°C for 1 min, and a final extension at 72°C for 2min.

The COI and RP‐15 amplicons of the expected size were cloned into the TA cloning vector pGEM‐T Easy (Promega, Madison, WI) by heat shock‐mediated transformation of *E*. *coli* DH5α competent cells (Green & Sambrook, 2012). Colony‐PCR amplification (Gussow & Clackson, 1989) was carried out using the M13F‐5'‐TGTAAAACGACGGCCAGT and M13R‐5'‐AGGAAACAGCTATGACCATG primers (Promega, Madison, WI) to identify clones containing the expected insert size. Cycling conditions were initial denaturation at 94°C for 10 min, 35 cycles at 94°C for 60 s, 53°C for 60 s and 72°C for 1 min, and final extension at 72°C for 10 min. Initially, one COI and two RP‐15 clones per whitefly sample were subjected to bi‐directional DNA Sanger sequencing. Each cloned insert was sequenced bidirectionally using an ABI 3700 capillary sequencer at the Genomics Core Facility, The University of Arizona, Tucson, Arizona, USA (http://uagc.arizona.edu).

To amplify additional RP‐15 alleles from the *B*. *tabaci* haplotypes, NAFME 8 (EN8_1 and EN8_3) and NAFME 3 (Eth3_1, and Eth3_2), respectively, 8–10 cloned RP‐15 amplicons were subjected to DNA sequencing as described above.

### 2.2 Pairwise distances and phylogenetic analysis

Sequences were aligned using MUSCLE v3.8.31 (Edgar, 2004), implemented in the Align Multiple Sequences tool available in Mesquite v2.75 (Maddison & Maddison, 2018). The mtCOI and RP‐15 sequence alignments were trimmed to 1022 and 754 nt in length, respectively, and terminal gaps were treated as missing data. The best‐fitting evolutionary models for the mtCOI and RP‐15 sequences were estimated based on consensus of the Bayesian information criterion, Akaike information criterion, and maximum likelihood value using jModelTest v2.1.10 (Darriba et al., 2012). The Hasegawa–Kishino–Yano model (Hasegawa et al., 1985) with gamma‐distributed rate variation among sites, and the Kimura‐2‐parameter model (Kimura, 1980) were identified as the best‐fit evolutionary model for the mtCOI and nuclear RP‐15 data, respectively. Corrected pairwise distances (sequence divergence) for mtCOI and RP‐15 sequences were calculated in PAUP v4.0a (Swofford, 2003).

The mtCOI sequences from Brazil samples and reference sequence EN8 (NAFME 8), showed minimal to no sequence divergence, therefore representative sequences from Brazil (100% nt similarity), 40339, 40348, 40375, 40399, 40400, and 40415, were selected for phylogenetic analysis. The mtCOI sequences representing *B*. *tabaci* exemplar haplotypes, NAFME 1–8 (Table S1) were included as references to resolve all eight previously recognized B mitotype haplotype groups (Paredes‐Montero et al., 2021). The nuclear RP‐15 sequences from Brazil samples, 40339, 40348, 40375, 40399, 40400, and 40415, were aligned with 8–10 RP‐15 sequences determined from *B*. *tabaci* from Israel (EN8_1 and 3) (NAFME 8) and Ethiopia (Eth3‐1 and 2) (NAFME3).

The mtCOI and RP‐15 sequences for the A mitotype (New World species) from Ecuador, and Q‐like mitotype from Uganda (Table S1), were included for between‐mitotype divergence comparisons. The mtCOI and RP‐15 phylogenetic trees were rooted with the mtCOI and RP‐15 sequence, respectively, of the “Uganda‐Sweet potato” *Bemisia* spp., collected from sweet potato plants in Namulonge, Uganda.

Bayesian analysis was carried out using MrBayes v3.2.6 (Huelsenbeck & Ronquist, 2001) consisting of four independent Markov chain Monte Carlo (MCMC) runs, each with four Markov chains, permitted to run for 1 × 10^6^ generations. Trees were sampled every 200th generation. Log‐likelihood scores were plotted with the generations sampled using Tracer v1.6 (Rambaut et al., 2018), and the effective sample size was confirmed to be >200. The MCMC runs were summarized using sump and sumt commands in MrBayes v3.2.5 (Huelsenbeck & Ronquist, 2001). Trees in the first 1 × 10^5^ generations per replicate were discarded as burn‐in. The majority‐rule consensus tree was drawn using FigTree v1.4.2 (http://tree.bio.ed.ac.uk/software/figtree/).

### 2.3 Simulations of demographic expansion of the B mitotype in Brazil

#### 2.3.1 Dispersal assumptions

Simulations of population expansion were estimated using SPLATCHE v3 (Currat et al., 2019) in a two‐dimensional stepping‐stone model defined on a matrix of ~27.5 × 10^4^ demes (pixels or cells) covering Brazil and parts of surrounding South American countries. Each deme represented a surface of ~72 km^2^ and exchanged migrants with its four neighbors depending on the pre‐defined parameters: (1) carrying capacity, an estimate of the maximum number of whiteflies that could be sustained given predicted resources available in the geographic deme; (2) friction, corresponding to the ease by which whiteflies move from deme to deme; and (3) migration rate, which refers to the percentage of the population likely to spread to a neighboring deme. Maps of land cover and topographic information for Brazil (altitude) were obtained from the geographical information system repository (https://www.diva‐gis.org/gdata). The altitude map was coded using distinct friction values for each cell using QGIS v 3.16.3 (http://qgis.osgeo.org). Maps were re‐sized to a layer resolution of 0.08 and exported as ASCII raster layers using the conversion tool available in the “Raster” menu, and unnecessary rows in map headers were removed using a text editor. The “land cover” map consisted of one of 22 vegetation types based on the classification by the Global Land Cover 2000 Project (GLC20000) (https://forobs.jrc.ec.europa.eu/products/glc2000/glc2000.php).

Carrying capacity and friction values were assigned to demes based on the quality of habitat and barriers to whitefly dispersal (e.g., altitudes of >600 m a.s.l.). For instance, the largest relative carrying capacities at 100%, were assigned to the following vegetation types: agriculture intensive, mosaic agriculture degraded forests, mosaic agriculture degraded vegetation. The lowest carrying capacity of 0% was assigned to micro‐niches with a vegetation type unlikely to sustain whitefly populations, which are fresh water flooded forests, mangroves, barren bare soil, water bodies, and permanent snow ice.

The likelihood of whitefly migration rate was given as a friction value that ranged from 0 to 1, where 0 was assigned to demes with no impediments for whitefly migration, that is, migration is strong, and 1 corresponded to extreme friction, or when migration is not possible. Friction type was set to 2 to consider barriers to dispersal given by the vegetation type (land cover) and altitude. For the vegetation map, the same criteria used to define carrying capacities were used to assign friction values, with the lowest friction assigned to irrigated agro‐ecosystems (Brown & Bird, 1992; Brown et al., 1995), consistent with the habitat in which the B mitotype is predicted to support rapid expansion through migration and dispersal (Bethke et al., 1991; Butler et al., 1989; Naranjo et al., 2010), and where the highest friction corresponded to ecosystems where *B*. *tabaci* was “least likely” to establish, for example, in bodies of water or dense, humid tropical forested areas, and other ecosystems that are inconsistent with known B mitotype habitats. For altitude, extreme friction was set to values above 600 m a.s.l. and lowland areas were set to anticipate unimpeded flight and dispersal.

A migration rate of 6% of the population at each deme was assigned in accordance with the migratory proportion of flight morphs identified for the *B*. *tabaci* Arizona‐B mitotype (Blackmer & Byrne, 1993; Byrne & Houck, 1990). In the simulation, westward dispersal was predicted for half of the migratory whitefly morphs, based on the predicted direction of flight (dispersal) in Brazil, predicted as, east to west (Gontijo et al., 2013), with the remaining proportion of migratory whiteflies evenly distributed among the other three possible directions of dispersal, excluding ecosystems where they were deemed unlikely to establish (Stansly & Naranjo, 2010).

#### 2.3.2 *B. tabaci* field and laboratory life tables

Indicators of reproduction, development, and survival were used to model the demographic expansion of the B mitotype in Brazil. Several studies have measured basic demographic parameters under controlled environments (Carabali et al., 2005; Kakimoto et al., 2007; Ma, 2005; Musa & Ren, 2005; Tsai & Wang, 1996), with the accepted caveat that these measures are expected to accurately reflect whitefly death occurrence or death rate in their natural habitats most but not necessarily 100% of the time. Laboratory demographic measures were compiled and corrected using the likelihood of survival estimated for agroecosystems of Brazil (de Albergaria et al., 2003). Given the variable net reproductive rate (R_0_) known to vary between host plants (average female offspring produced per female), the simulation considered minimum, maximum, and average R_0_ values of 19.75, 158.24, and 54.30, respectively. The values were corrected (Rc_0_) using the observed probability of survival in soybean fields of Brazil at 0.177 (de Albergaria et al., 2003). SPLATCHE software considers the intrinsic rate of natural increase of a population to make demographic predictions, obtained by dividing the natural logarithm of the Rc_0_ by the generation time of ~25 days in the tropical and subtropical regions of Brazil (Faria et al., 2016; Mansaray & Sundufu, 2009). The demographic model was selected to account for one population to engage in long‐distance dispersal. Simulations were carried out for 30 generations with ASCII raster layers created after each 5‐generations to predict the relative density of the B mitotype in each deme occupied. The minimum, maximum, and average simulations determined for the two oldest archived collections of the B mitotype available, which were Rio de Janeiro (1989) and Ceará (1990), respectively, were merged in QGIS v 3.16.3 (http://qgis.osgeo.org). The depictions are considered predictors of demographic expansion of the B mitotype, assuming arrival, establishment, and spread from the different predicted sites of introduction in Brazil.

## 3 RESULTS

### 3.1 Mitotype identification by mtCOI and RP‐15 sequence analyses

**FIGURE 1:**
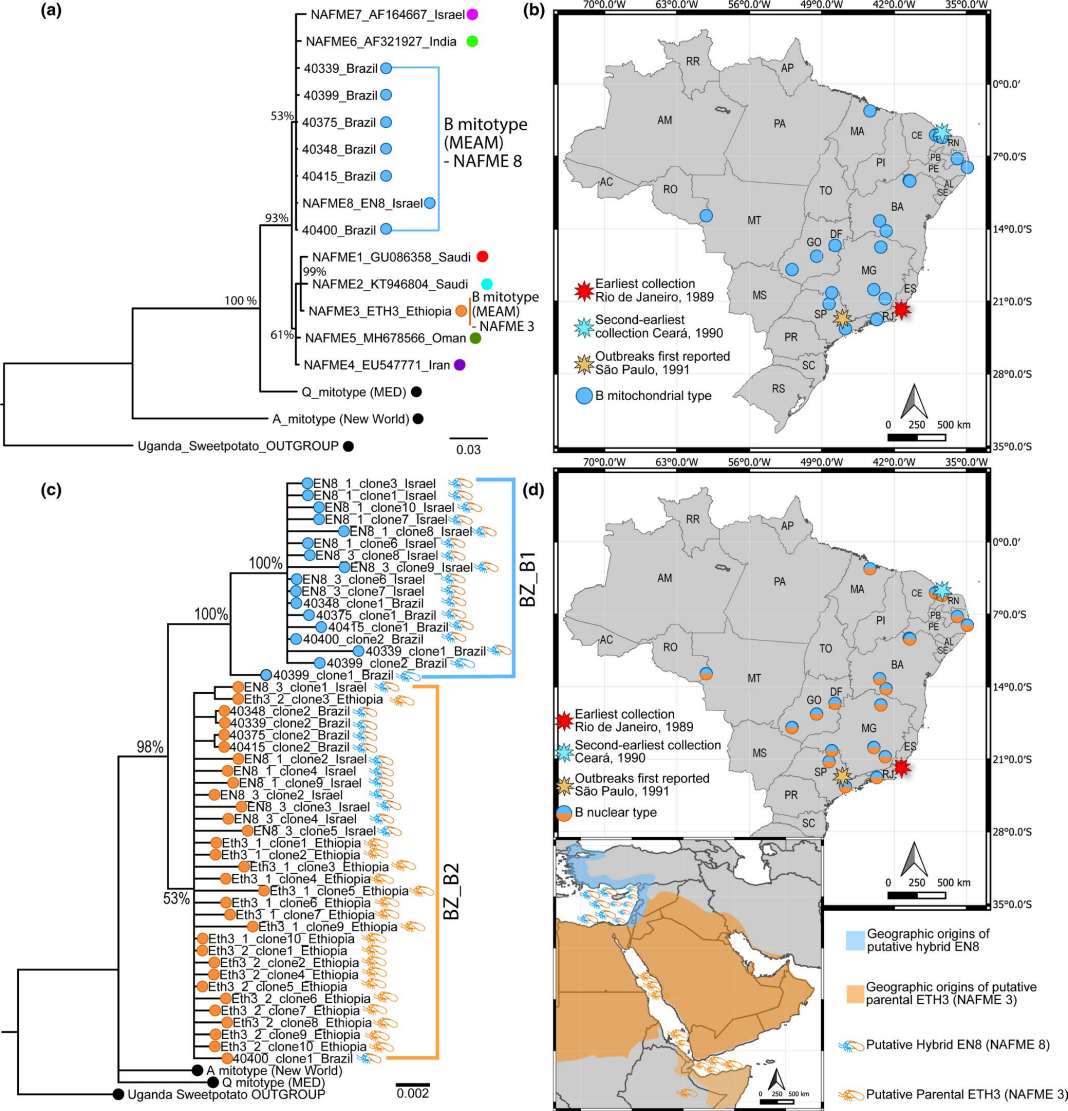
Phylogenies and distribution of *Bemisia tabaci* B haplotypes in Brazil. (a) Bayesian mtCOI phylogenetic tree showing non‐redundant representative sequences of collections from 1989 to 2005. (b) Distribution of the B mitochondrial type in Brazil. (c) Bayesian RP‐15 phylogeny resolved two diverging nuclear clades, BZ‐B1 and BZ‐B2 in female *B*. *tabaci* collected in Brazil during 1989–2005. Only non‐redundant representative nuclear sequences were included in the tree. Colored whitefly cartoons show the position of reference samples EN8 (NAFME 8) and Eth3 (NAFME 3) in the phylogenetic tree. (d) Distribution of putative *B*. *tabaci* hybrids in Brazil, lower‐left map shows estimated niche ranges of EN8 (NAFME 8) and Eth3 (NAFME 3) whitefly references in their center of origin. Node support corresponded to posterior probabilities. The sequences of a Uganda‐Sweet potato *B*. *tabaci* mitotype was used to root phylogenetic trees

Based on comparative mtCOI sequence analysis, the *B*. *tabaci* whiteflies collected from 1989 to 2005 in Brazil were identified as haplotype NAFME 8 of the B mitotype, most closely related to the Israel B‐like reference (EN8, NAFME 8). Results of the mtCOI pairwise distance analysis indicated that the *B*. *tabaci* B‐mitotype sequences from Brazil diverged from those of the Ethiopia 3 (Eth3, NAFME 3) and the NAFME 1–7 haplotypes by 1.8% and 0.5–1.9%, respectively (Figure 1a,b).

Phylogenetic analysis indicated the Brazil mtCOI sequences grouped with haplotypes 6–8 of the NAFME clade, which clustered as a sister clade to the NAFME haplotypes 1–5 (Figure 1a). The NAFME sister clades were monophyletic with the Q mitotype reference mtCOI sequence from Uganda, to form the NAF‐MED‐ME lineage (de Moya et al., 2019). As expected, the A mitotype and Uganda‐Sweet potato reference mtCOI sequences, representing distinct cryptic species, grouped as outliers.

The oldest B mitotype record in the EMBRAPA whitefly collection studied herein was represented by a sample collected in 1989 in Rio de Janeiro, southeastern Brazil. The second‐earliest record was represented by a sample collected in 1990 in Ceará state in northeastern Brazil on the Atlantic coast (Figure 1b). These results suggest that the outbreaks of B mitotype reported during 1991 in São Paulo city (São Paulo state) (Lourenção & Nagai, 1994) were likely associated with invasions associated with westward‐dispersing *B*. *tabaci* originating from Rio de Janeiro where they were introduced on infested ornamental plants.

Based on phylogenetic analysis of the RP‐15 nuclear sequences, females (2*n*) of the B mitotype analyzed from the 1989–2005 collections were found to harbor two different alleles, which corresponded to the two well‐differentiated BZ‐B1 and BZ‐B2 subclades (Figure 1c). In addition, one *B*. *tabaci* sample, which was represented by a single male (haploid) (40399), yielded one allele, inadvertently serving as an internal control. The B mitotype (female) endemic to Israel (EN8, NAFME 8) also harbored BZ‐B1 and two alleles. In contrast, the nuclear RP‐15 sequences determined for the reference Eth3 (NAFME 3) (female) from Ethiopia, grouped with the BZ‐B2 subclade only.

The 1.5% intra‐mitotypic divergence between the BZ‐B1 and BZ‐B2 haplotypes was similar to the inter‐mitotypic divergence between the BZ‐B1 and BZ‐B2 and the A‐mitotype (cryptic species) reference, at 1.5–2.2% pairwise distance, which was 0.1% less than the pairwise distances between the B and Q nuclear types, at 1.6–1.75%.

Collectively, these results suggest that the invasive B haplotype associated with the outbreaks in Brazil, identified as NAFME 8, and the reference EN8 (NAFME 8) from Israel, could possibly represent the offspring resulting from a potentially recent hybridization event. Further, results indicate that one parent of hybrid NAFME 8 is most closely related to the B mitotype reference from Ethiopia (NAFME 1–3). The other parental allele(s) did not match any of the RP‐15 alleles determined here and further exploration was beyond the scope of this study (Figure 1c,d).

### 3.2 Simulations of demographic expansion of the B mitotype in Brazil

**FIGURE 2:**
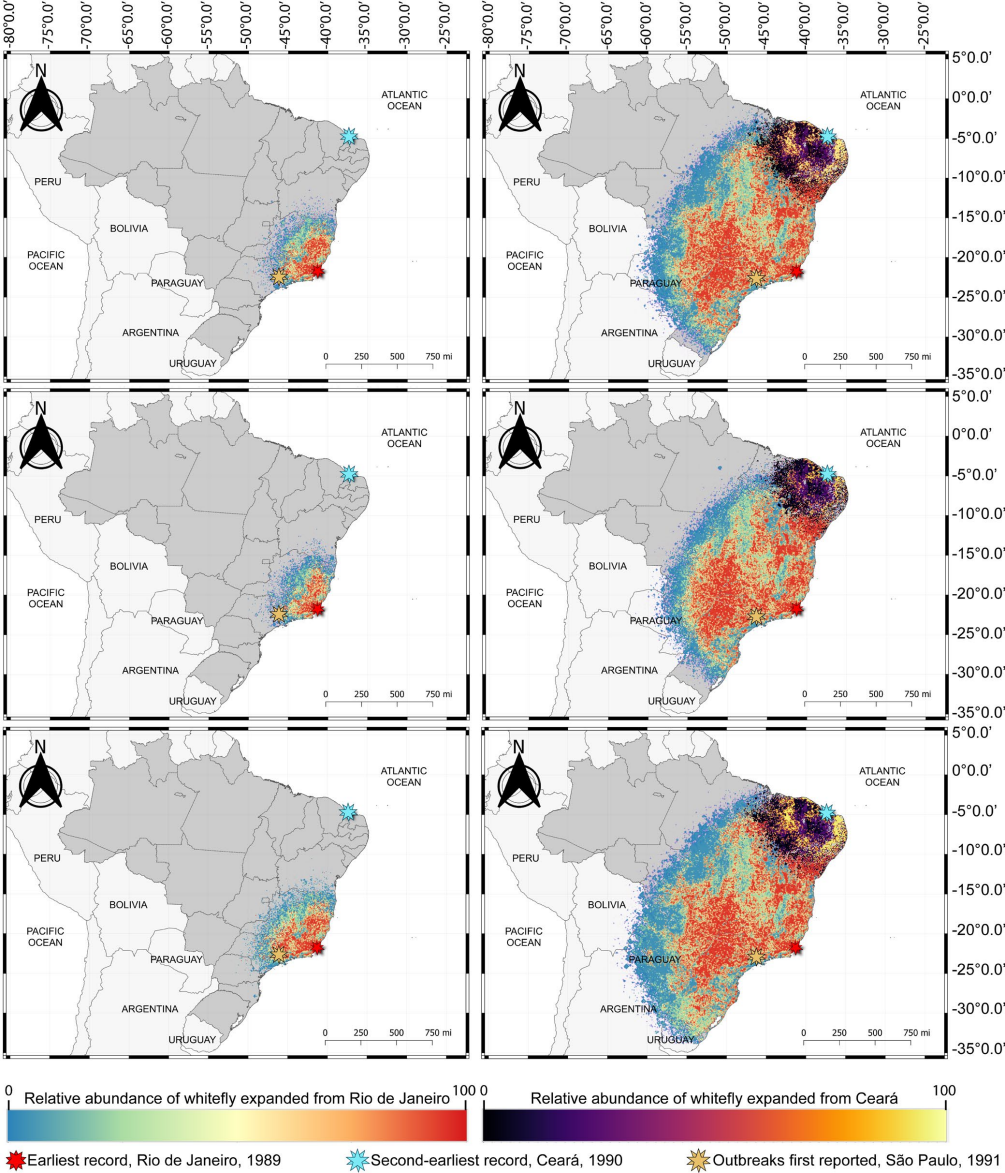
SPLATCHE simulations of demographic expansion of the *Bemisia tabaci* B mitotype in Brazil. Left‐upper, ‐middle and ‐lower maps show the simulated expansion of the B mitotype from Rio de Janeiro after 15 generations using minimum, average and maximum demographic parameters, respectively. Right‐upper, ‐middle and ‐lower maps show the simulated expansion of the B mitotype from Rio de Janeiro after 30 generations using minimum, average and maximum demographic parameters, respectively, and from Ceará after 15 generations

The demographic expansion predictions outward from Rio de Janeiro, the earliest record of the B mitotype presence, indicated that the B mitotype likely dispersed westwardly, unimpeded, at a speed rate of 200–500 km/year, reaching São Paulo in only 15 generations by 1990, but would have been unable in this same year to reach Ceará, the site of second oldest archived B mitotype sample (record) in Brazil (Figure 2). The low‐friction migration micro‐niches in the tropical semideciduous forest and the “cerrado” (savanna) eco‐regions with extensive monocrop acreage appear to have facilitated the rapid spread of B mitotype populations from expansions emanating from Rio de Janeiro. These analyses predicted that outbreaks of the B mitotype reported in São Paulo during 1991 (Lourenção & Nagai, 1994) were linked to the westward‐dispersing populations originating in Rio de Janeiro.

Simulations of the demographic expansion from Ceará, the second‐earliest record of the B mitotype (this report), indicated that populations invading from this locale migrated at a slower rate than the those from Rio de Janeiro, at 100–300 km/year. The slower rate can likely be attributed to the climatic conditions there, which are unfavorable for *B*. *tabaci* to establish, consisting of micro‐niches in the Caatinga and transitional palm forest that surrounds Icapuí, Ceará, or “tropical vegetation,” from where *B*. *tabaci* samples were collected in 1990 (Figure 2). Based on the archived whitefly samples collected from Ceará in northeastern Brazil, this site corresponds to a second independent B mitotype introduction that with populations expanding and dispersing from Rio de Janeiro, are predicted to have completed the invasion of Brazil in only 30 generations (Figure 2).

### 3.3 Plant species associated with early B mitotype collections in Brazil

The plant species binomial names were corroborated using the botanical database Tropicos^®^ (https://www.tropicos.org). The *B*. *tabaci* samples were collected during 1989–2005 from the following eight plant species, broccoli *Brassica oleracea* var. *italica* Plenk (*n* = 4), cabbage *B*. *oleracea* var. *capitata* L. (Brassicaceae) (*n* = 5), cucumber *Cucumis sativus* L. (*n* = 2), muskmelon *C*. *melo* L. (Cucurbitaceae) (*n* = 4), soybean *Glycine max* L. (Fabaceae) (*n* = 2), wild *Solanum* spp. (Solanaceae) (*n* = 5), unidentified *Cucurbita* spp. (Cucurbitaceae) (*n* = 2), unidentified weed species (*n* = 4), followed by one observation on *Abelmoschus esculentus* (L.) Moench (Malvaceae), *Citrullus lanatus* (Thunb.) Mansf. (Cucurbitaceae), *Gossypium* sp. (Malvaceae), *Solanum lycopersicum* (L.) H. Karst. (Solanaceae), *Nicotiana tabacum* L. (Solanaceae), *Phaseolus* sp., *P*. *vulgaris* L. (Fabaceae), and *Spondias mombin* L. (Anacardiaceae).

The 1989 Rio de Janeiro sample (southeast region), the oldest known museum archive of the *B*. *tabaci* B mitotype in Brazil, was collected from broccoli plants, *B*. *oleracea* var *italica*, one of several host plant species not preferred by *B*. *tabaci* endemic to Brazil or elsewhere in the Americas (Brown, 2010; Brown et al., 1992). The B mitotype sample archived in 1990 from Ceará state was collected from *Spondias mombin*, or hog plum, which is native to the tropical Americas, including the Eastern Caribbean region. Because Anacardiaceous species have not been known to serve as reproductive hosts of *B*. *tabaci*, it is most likely that the infestations of *S*. *mombin* were associated with populations dispersing from densely colonized preferred host plants including nearby senescing crops, even though reproduction on *S*. *mombin* was unlikely to occur.

## 4 DISCUSSION

Although the B‐mitotype was first implicated in whitefly *B*. *tabaci* outbreaks in São Paulo, Brazil based on the silver‐leaf symptoms observed there in pumpkin plants (Lourenção & Nagai, 1994), the precise location or year of the proposed introduction had not been substantiated by genetic studies. Here, *B*. *tabaci* field samples collected from different regions and plants species during 1989–2005 were identified by comparative sequence analysis with a mtCOI reference database (JK Brown Lab, University of Arizona), and genotyped using the RP‐15 gene as a nuclear marker (Figure 1). Results of comparative mtCOI sequence analysis identified the 35 *B*. *tabaci* archived field collections as the NAFME 8 haplotype of the B mitotype with near‐zero intra‐mitotypic variation. Here, the first introduction of the B mitotype in Brazil was found to have occurred in 1989 in Campos dos Goytacazes, a coastal city in Rio de Janeiro. The following year another introduction took place in the coastal city Icapuí, in Ceará, which is located near Mossoró (Rio Grande do Norte State), a region where melon was reported to be heavily infested by *B*. *tabaci* resulting in feeding damage and outbreaks of whitefly‐transmitted viruses (Lima et al., 2000; Queiroz et al., 2017). Identification of these two locations as the sites of the earliest introductions of the B mitotype is feasible because both locales are in proximity to the major commercial ports of entry through which ornamentals, exotic plants, and fruits are regularly imported, with the Atlantic seaports receiving the heaviest flow of plants and plant‐related goods.

Further, the year of collection for the whitefly samples analyzed in this study are without question because they represent subsets of specimens archived in the EMBRAPA insect collection before the purported 1991 outbreak, which was not reported until three years later, in 1994 (Lourenção & Nagai, 1994). That the B mitotype was introduced into Brazil for the first time in 1989, is consistent with the earliest observations of silverleaf symptoms in squash throughout Central America and Caribbean region during 1988–1990 (Brown, 1994; Brown & Bird, 1992; Costa & Brown, 1990), and only slightly later in South America (Viscarret et al., 2003).

Simulations of whitefly migrations suggested the *B*. *tabaci* colonization begun in Rio de Janeiro in 1989 followed by an unprecedented rapid barrier‐free migration facilitated by the predominant monocropping systems in the “Cerrado” savanna, an eco‐region whose plant community is expected to have aided in the dispersal of the B mitotype. Despite the simulated migration rate of 200–500 km/year, the predicted expansion of the B mitotype founder population from Rio de Janeiro could not feasibly account for the dispersal of subsequent generations to Ceará by 1990. Therefore, the 1989 and 1990 records of the B mitotype in Brazil represent independent introductions.

The two earliest detections of the B mitotype were predicted to be capable of invading and establishing throughout most of tropical–subtropical Brazil, which has an estimated surface area of 2,100,000 mi^2^ (excluding the Amazon rainforest), in 2 years or less. The rapid geographic expansion of *B*. *tabaci* from the two independent introductions, traceable to 1989 and 1990, respectively, led to the two migrating populations to coalesce and establish a nearly country‐wide infestation by ~1991. By comparison, invasion of the US by the B mitotype, including the suspect initial sites of introduction first in Hawaii and then in Florida, required approximately 4–5 years (1986–1991) (Bellows et al., 1994; Brown, Coats, et al., 1995; Gill & Brown, 2009), whereas, complete establishment of the B mitotype in North Queensland, with a surface area of 30,900 mi^2^, is estimated at 3–4 years (Liu et al., 2007). Regular surveillance of *B*. *tabaci* in Brazil and elsewhere in the Tropical Americas would facilitate the early detection of other exotic *B*. *tabaci* haplotypes, while also inform pest management strategies to prevent crop loss due to damage caused by *B*. *tabaci* as a pest and vector of plant viruses.

Unexpectedly, analysis of the RP‐15 nuclear marker revealed that the invasive haplotype of the B mitotype in Brazil and a reference population from Israel, EN8 (NAFME 8), harbored two alleles, BZ‐B1 and BZ‐B2. Further, only one of the two alleles, BZ‐B2, was recovered from the Eth3 reference (NAFME 3) from Ethiopia, suggesting the B haplotype that invaded Brazil (and elsewhere) represents a potentially recent, intra‐specific hybridization event. The NAFME region is the predicted center of origin of the B mitotype (Brown, 2010), and recently, the B mitotype has been shown to comprise at least eight phylogeographic haplotypes, several that inhabit overlapping niche ranges with potential as “hybrid zones” (Paredes‐Montero et al., 2021). The B‐mitotypes, EN8 (NAFME 8) and Eth3 (NAFME 3), are two examples of such haplotypes. Thus, in one hybridization scenario, the Eth3 (NAFME 3) or closely related NAFME 1–2, harboring only one of the two RP‐15 alleles, served as one of the two parental haplotypes. The EN8 (NAFME 8) haplotype, which carries two different RP‐15 alleles, could feasibly be the founder (hybrid) population of the B mitotype that has been introduced worldwide (Paredes‐Montero et al., 2021). Although the other parent haplotype has not been identified among the reference samples analyzed here (albeit, a small sample size), the missing parent could have been displaced by its hybrid offspring (Abbott et al., 2016; Bellows et al., 1994; Chaturvedi et al., 2020; Epifanio & Philipp, 2000). This latter hypothesis offers the most parsimonious explanation, given the substantial knowledge of its invasiveness and extensive damage to food, fiber, and ornamental crops since the earliest introductions, which occurred during the 1980s. In addition, NAFME 8 is known to displace at least two mitotypes native to the Americas (New World A, Jatropha) and/or exotic or endemic Q haplotype(s) (Brown, 2010; Brown, Coats, et al., 1995; Liu et al., 2007; Paredes‐Montero et al., 2020; Zang et al., 2005). Finally, the co‐existence of putative hybrids and parental taxa, including Eth3 (NAFME 3) or its closest relative (NAFME 1–2), could be explained based on the diverse climate niches they occupy, spanning the Arabian Peninsula to portions of coastal northeast Africa (Hadjistylli, 2010; Paredes‐Montero et al., 2021).

The fixation of one mitochondrial type in the putative intra‐specific hybrid, EN8 (NAFME 8), suggests the possibility of unidirectional gene flow as a potential driver of selection that favored the hybridization event. Unidirectional gene flow is well‐known to be associated with increased competitive advantage of a dominant parent over a less dominant one, which can result in asymmetric mating (Caballero, 2007; Liu et al., 2007). Alternatively, unidirectional hybridization can be explained by cytoplasmic incompatibility (Nirgianaki et al., 2003; Li et al., 2007; Lv et al., 2021) phenotypes such as male‐killing (Jiggins, 2003; Lv et al., 2021; Pan et al., 2012), and feminization of genetic males, which have been attributed to endosymbionts known to be harbored by whiteflies, including *Rickettsia*, *Wolbachia*, and *Cardinium* (Barro & Hart, 2000; Caballero, 2007; Giorgini et al., 2009). An expanded analysis by whole genome sequencing of extant B haplotypes could provide important new insights into the nature of this hybrid, NAFME 8, which has been ostensibly referred to as “superbug” (Kays et al., 1994; Markham et al., 1994; Perring, 2001; Wan et al., 2009).

## CONFLICT OF INTEREST

The authors declare no conflict of interest.

## AUTHOR CONTRIBUTIONS

**Jorge R. Paredes‐Montero:** Conceptualization (equal); Data curation (equal); Formal analysis (equal); Investigation (equal); Methodology (equal); Software (equal); Visualization (equal); Writing – original draft (equal); Writing – review & editing (equal). **Muriel Rizental:** Conceptualization (equal); Data curation (equal); Formal analysis (equal); Investigation (equal); Methodology (equal); Writing – original draft (equal). **Eliane Dias Quintela:** Conceptualization (equal); Funding acquisition (equal); Investigation (equal); Methodology (equal); Project administration (equal); Resources (equal); Supervision (equal); Validation (equal); Writing – review & editing (equal). **Aluana Gonçalves de Abreu:** Conceptualization (equal); Data curation (equal); Investigation (equal); Methodology (equal); Resources (equal); Supervision (equal); Writing – review & editing (equal). **Judith K. Brown:** Conceptualization (lead); Funding acquisition (equal); Investigation (equal); Methodology (equal); Project administration (equal); Resources (equal); Supervision (equal); Validation (equal); Writing – original draft (equal); Writing – review & editing (equal).

## Supporting information

Table S1

## Data Availability

The mtCOI and RP‐15 sequences were submitted to GenBank, and accession numbers are available in Table S1, which was deposited to Dryad at the following URL: https://doi.org/10.5061/dryad.41ns1rnfz.
