# Supplementary material for: Earlier than expected introductions of the Bemisia tabaci B mitotype in Brazil reveal an unprecedented, rapid invasion history
Source: Ecol Evol. 2022 Jan 23;12(1):e8557. doi: 10.1002/ece3.8557 (PMC8796915; doi:10.1002/ece3.8557)
Supplement: Supplementary file 1 — Table S1 [file ECE3-12-e8557-s001.docx]

**Earlier than expected introductions of the *Bemisia tabaci* B mitotype in Brazil reveal an unprecedented, rapid invasion history**

**Jorge R. Paredes-Montero ^1,2,¥^, Muriel Rizental ^3,4,¥^, Eliane Quintela ^4^, Aluana Gonçalves Abreu^4^, Judith K. Brown ^1*^**

^1^ School of Plant Sciences, The University of Arizona, Tucson, AZ 85721 USA

^2^ Facultad de Ciencias de la Vida, Escuela Superior Politécnica del Litoral, ESPOL, Campus Gustavo Galindo Km 30.5 Vía Perimetral, P.O. Box 09-01-5863, Guayaquil, Ecuador

^3^ Doctoral Student, Federal University of Goiás, Goiânia, GO, 74690-900, Brazil

^4^ EMBRAPA Rice and Beans. Rodovia GO-462, Km 12, Zona Rural, CP. 179, Santo Antônio de Goiás, GO, 75375-000, Brazil

| **Supplementary Table 1** | | | | | | | | | |
| --- | --- | --- | --- | --- | --- | --- | --- | --- | --- |
| **Code** | **Location** | **Latitude** | **Longitude** | **Collection date** | **Host plant** | **GenBank Accession Numbers** | | | |
|  |  |  |  |  |  | **mtCOI** | **RP-15** | | |
|  |  |  |  |  |  |  | **BZ-B1** | **BZ-B2** | **Outgroups** |
|  | **North** |  |  |  |  |  |  |  |  |
|  | **RONDÔNIA (RO)** |  |  |  |  |  |  |  |  |
| 40345 | Vilhena | -12.734487 | -60.1611 | 2004 | *Phaseolus vulgaris* |  |  |  |  |
|  | **NORTHEAST** |  |  |  |  |  |  |  |  |
|  | **BAHIA (BA)** |  |  |  |  |  |  |  |  |
| 40399 | Bom Jesus da Lapa | -13.23628 | -43.40029 | 1999 | *Cucurbita sp.* | MZ726399 | MZ733457 |  |  |
| 40420 | Guanambi | -14.204061 | -42.776993 | 1997 | *Gossypium sp.* |  |  |  |  |
|  | **CEARÁ (CE)** |  |  |  |  |  |  |  |  |
| 40359 | Icapuí | -4.71523 | -37.354173 | 1990 | *Spondias mombin* | MZ726395 |  |  |  |
| 40398 | Russas | -4.937551 | -37.997103 | 1997 | *Cucumis melo* |  |  |  |  |
|  | **MARANHÃO (MA)** |  |  |  |  |  |  |  |  |
| 40397 | São Luís | -2.611004 | -44.338454 | 2005 | *Cucumis sativus* |  |  |  |  |
|  | **PARAÍBA (PB)** |  |  |  |  |  |  |  |  |
| 40375 | Campina Grande | -7.225.764 | -35.903964 | 2005 | *Gossypium sp.* | MZ726393 | MZ733460 | MZ733466 |  |
|  | **PERNAMBUCO (PE)** |  |  |  |  |  |  |  |  |
| 40380 | Petrolina | -9.325694 | -40.55039 | 2000 | *Solanum sp.* |  |  |  |  |
| 40395 | Petrolina | -9.387342 | -40.487729 | 2003 | *Citrullus lanatus* |  |  |  |  |
| 40417 | Petrolina | -9.387342 | -40.487729 | 2003 | *Cucurbita sp.* |  |  |  |  |
| 40384 | Petrolina | -9.387342 | -40.487729 | 2003 | *Solanum sp.* |  |  |  |  |
| 40383 | Recife | -8.069346 | -34.922491 | 1999 | *Brassica oleracea* | MZ726392 |  |  |  |
|  | **RIO GRANDE DO NORTE (RN)** |  |  |  |  |  |  |  |  |
| 40339 | Baraúna | -5.080659 | -37.620628 | 2001 | *Cucumis melo* | MZ726398 | MZ733458 | MZ733463 |  |
| 40337 | Mossoró | -5.17301 | -37.345074 | 2000 | *Cucumis melo* |  |  |  |  |
| 40348 | Mossoró | -5.17301 | -37.345074 | 2000 | *Weed* | MZ726391 | MZ733462 | MZ733464 |  |
| 40340 | Mossoró | -5.17301 | -37.345074 | 2000 | *-* |  |  |  |  |
| 40368 | Mossoró | -5.17301 | -37.345074 | 2000 | *Cucumis melo* |  |  |  |  |
|  | **MIDWEST** |  |  |  |  |  |  |  |  |
|  | **DISTRITO FEDERAL (DF)** |  |  |  |  |  |  |  |  |
| 40335 | Brasília | -15.604142 | -47.712883 | 2003 | *Nicotiana tabacum* |  |  |  |  |
| 40338 | Brasília | -15.604142 | -47.712883 | 2003 | *Brassica oleracea* |  |  |  |  |
| 40358 | Brasília | -15.603499 | -47.713773 | 2003 | *Brassica oleracea* |  |  |  |  |
| 40363 | Brasília | -15.603499 | -47.713773 | 2003 | *Abelmoschus esculentus* |  |  |  |  |
| 40365 | Brasília | -15.603499 | -47.713773 | 2003 | *Weed* |  |  |  |  |
| 40419 | Brasília | -15.603499 | -47.713773 | 2003 | *Brassica oleracea var. italica* |  |  |  |  |
| 40356 | Brasília | -15.603499 | -47.713773 | 2003 | *Brassica oleracea* |  |  |  |  |
|  | **GOIÁS (GO)** |  |  |  |  |  |  |  |  |
| 40361 | Jataí | -17.938925 | -51.871001 | 2003 | *Solanum sp.* |  |  |  |  |
| 40382 | Trindade | -16.645978 | -49.496877 | 1999 | *Lycopersicon lycopersicum* |  |  |  |  |
| 40400 | Trindade | -16.645978 | -49.496877 | 1999 | *Solanum sp.* | MZ726396 | MZ733461 | MZ733467 |  |
|  | **SOUTH EAST** |  |  |  |  |  |  |  |  |
|  | **MINAS GERAIS (MG)** |  |  |  |  |  |  |  |  |
| 40415 | Belo Horizonte | -19.871055 | -43.97 | 1999 | *Solanum sp.* | MZ726397 | MZ733459 | MZ733465 |  |
| 40374 | Nova Porteirinha | -15.766.366 | -43.286335 | 2000 | *Ipomoea batatas* |  |  |  |  |
| 40376 | Viçosa | -20.75835 | -42.868366 | 1998 | *Glycine sp.* |  |  |  |  |
|  | **RIO DE JANEIRO (RJ)** |  |  |  |  |  |  |  |  |
| 40416 | Seropédica | -22.772021 | -43.689456 | 2003 | *Brassica oleracea* |  |  |  |  |
| 40369 | Campos dos Goytacazes | -21.749602 | -41.3087 | 1989 | *Brassica oleracea var. italica* | MZ726394 |  |  |  |
|  | **SÃO PAULO (SP)** |  |  |  |  |  |  |  |  |
| 40367 | Jaboticabal | -21.246029 | -48.293697 | 1999 | *Cucumis sativus* |  |  |  |  |
| 40381 | Santo Amaro | -23.650978 | -46.710598 | 2000 | *Brassica oleracea var. italica* |  |  |  |  |
| 40418 | Miguelópolis | -20.174762 | -48.029275 | 1998 | *Glycine sp.* |  |  |  |  |
|  | **REFERENCES** |  |  |  |  |  |  |  |  |
| EN8 (NAFME 8) | Israel |  |  | 1994 | *Gossypium hirsitum* | MZ726389 | MZ733468 | MZ733469 |  |
| ETH3 (NAFME 3) | Ethiopia |  |  | 2004 | *Gossypium hirsitum* | MZ726390 |  | MZ733470 |  |
| Q mitotype | Uganda |  |  | 2006 | *Manihot esculenta* | MZ726400 |  |  | MZ733472 |
| AMTROP Lineage (New World) | Ecuador |  |  | 2014 | *Manihot esculenta* | MZ726401 |  |  | MZ733471 |
| Uganda Sweet-potato OUT-GROUP | Uganda |  |  | 2006 | *Ipomoea batatas* | MZ726402 |  |  | MZ733473 |
| NAFME 1 | Saudi Arabia |  |  |  |  | GU086358 |  |  |  |
| NAFME 2 | Saudi Arabia |  |  |  |  | KT946804 |  |  |  |
| NAFME 4 | Iran |  |  |  |  | EU547771 |  |  |  |
| NAFME 5 | Oman |  |  |  |  | MH678566 |  |  |  |
| NAFME 6 | India |  |  |  |  | AF321927 |  |  |  |
| NAFME 7 | Israel |  |  |  |  | AF164767 |  |  |  |
